# Veterinary Naturopathy and Complementary Medicine: A Survey among Homepages of German Veterinary Practitioners

**DOI:** 10.1159/000529209

**Published:** 2023-01-18

**Authors:** Ines Stanossek, Axel Wehrend

**Affiliations:** ^a^Kleintierklinik Frank, Freiburg, Germany; ^b^Justus-Liebig-University Gießen, Clinic for Obstretics, Gynaecology and Andrology for Large and Small Animal Science with Veterinary Ambulance, Giessen, Germany

**Keywords:** Complementary veterinary medicine, Veterinary naturopathy, Homeopathy, Phytotherapy, Acupuncture, Komplementärmedizin, Naturheilkunde, Homöopathie, Phytotherapie, Akupunktur

## Abstract

**Background:**

The use and interest in veterinary naturopathy and complementary medicine are increasing and modes of treatment are diverse. To this point, only a few data on the German situation in small animal medicine exist.

**Material and Methods:**

An online research of 1,083 German veterinary homepages for contents of veterinary naturopathy and complementary medicine was performed in September and October 2017. “Veterinarian” and “Chamber of Veterinary Surgeons” were used as search items. Homepages of small animal medicine were included. They were surveyed for modes of complementary and naturopathic treatments and corresponding qualifications of the offering veterinarian. Data were collected and processed using Excel 2013 Version 15.0. Afterward, a descriptive data analysis was performed.

**Results:**

60.7% (*n* = 657) of homepages showed contents of veterinary naturopathy and complementary medicine. The highest percentage was found in the Chamber of Veterinary Surgeons of Saarland (91.7%, *n* = 11 out of 12). Homeopathy was cited most frequently (58%, *n* = 381). Out of all homepages with relevant content, 31.4% (*n* = 206) gave information about user qualifications, with continuous education programs named most frequently (52.9%, *n* = 109).

**Conclusion:**

The given data illustrate the high number of German veterinary homepages with contents of veterinary naturopathy and complementary medicine, corresponding to actual data of a high usage in veterinary and human medicine. Therefore, further scientific research in this field seems reasonable. Modes of treatment and qualifications are highly diverse and despite of controversial public discussions, homeopathy was the most frequently cited treatment modality on German veterinary homepages.

## Zusammenfassung

**Hintergrund:**

Das Interesse und die Nutzung von Naturheilkunde und Komplementärmedizin in der Tiermedizin steigen und ihre Behandlungsverfahren sind vielfältig. Zum jetzigen Zeitpunkt liegen jedoch kaum deutsche Daten zur Situation in der Kleintiermedizin vor.

**Material und Methoden:**

Von September bis Oktober 2017 wurden 1,083 Homepages deutscher niedergelassener Tierärzte im Bereich Kleintiermedizin auf naturheilkundliche und komplementärmedizinische Inhalte untersucht. Mit den Suchbegriffen „Tierarzt/Tierärztin” und „Landestierärztekammer” wurden die entsprechenden Internetseiten aufgelisted. Nur Homepages mit Angaben zur Behandlung von Kleintieren wurden eingeschlossen. Erfasst wurden Behandlungsmodalitäten und angegebene Qualifikationen. Die Daten wurden mittels Excel 2013 Version 15.0 erfasst und im Anschluss deskriptiv analysiert.

**Ergebnisse:**

60.7% (*n* = 657) aller erfassten Homepages wiesen naturheilkundliche und komplementärmedizinische Inhalte auf, mit dem höchsten Anteil im Bereich der Landestierärztekammer Saarland (91.7%, *n* = 11/12). Homöopathische Inhalte wurden am häufigsten gefunden (58%, *n* = 381). Von allen Homepages mit relevanten Inhalten, machten 31.4% (*n* = 206/657) Angaben bezüglich vorhandener Qualifikationen. Hiervon wurden mit 52.9% (*n* = 109/206) am häufigsten weiterführende Fortbildungen genannt.

**Schlussfolgerung:**

Die vorliegende Studie zeigt eine hohe Anzahl an Homepages mit naturheilkundlichen und komplementärmedizischen Inhalten in der deutschen Kleintiermedizin. Diese Daten korrelieren mit jenen zur tatsächlichen Anwendungssituation in der Veterinär- und Humanmedizin. Die angewendeten Verfahren und vorhandenen Qualifikationen sind höchst vielfältig. Die Homöopathie, als in der Öffentlichkeit sehr häufig diskutiertes Verfahren, findet dabei die auffallend häufigste Erwähnung. Weitere Daten zur Erfassung der teils unübersichtlichen Situation werden gebraucht.

## Introduction

The use and interest in veterinary naturopathy and complementary medicine in Germany are increasing [1, 2], comparable to the situation in human medicine [3, 4, 5, 6] and other countries [7, 8]. Modes of treatment are diverse and face well-informed owners.

In the literature, different umbrella terms such as integrative, traditional, biological, complementary, or alternative medicine can be found. The World Health Organization (WHO) defines complementary medicine as treatment options which are not part of the countries own medical system [9]. Therefore, included treatment modalities can vary between countries. Furthermore, treatment modalities are not always categorized clearly. The different existing terms, definitions and organizations, including the lack of university structures, can be challenging for the usage of defined terms [10, 11]. Furthermore, terms and definitions evolve over time.

In the German literature of Stock-Schröer from 2013 [12] and Kraft and Stange from 2010 [13] and in comparison with other (veterinary) literature, complementary medicine includes homeopathy, homotoxicology, TCM (including acupuncture and Chinese herbs), neural therapy, organotherapy, and Bach Flower Remedies [7, 8, 14, 15, 16, 17, 18, 19, 20, 21, 22].

Especially in Germany (veterinary), naturopathy was historically separated from (veterinary) complementary medicine [10, 12, 13]. Modalities of naturopathy include phytotherapy, hydro-, electro and thermal therapy, hirudotherapy (diverting therapies), ostheopathy, and chiropractic [12, 13, 23, 24, 25, 26].

Lana et al. (2006) showed veterinarians to be the most frequently named resource of pet owners seeking for information about veterinary complementary medicine, regarding the treatment of their pets with cancer [18]. Despite that, there are only a few studies on the actual situation in small animal science in Germany. Some data can be found in the study of Hahn et al. [2] for the use of phytotherapy in German-speaking countries (Germany, Switzerland, Austria). With 79% they showed high numbers of usage, even though the investigated group of 189 respondents was relatively small [2]. In a previously published German questionnaire study of the authors, 85.4% of 795 small animal veterinarians used naturopathy and/or complementary veterinary medicine, noting the bias of active study participation [27].

In this context, this study aimed to give an insight into the actual situation in Germany by evaluating the presence of veterinary naturopathy and complementary medicine on homepages of German small animal practitioners. It explored cited treatment modalities and related qualifications of veterinarians offering these modalities.

## Material and Methods

An online research of 1,083 German homepages of veterinary practitioners was performed from 14th of September 2017 to 14th of October 2017. Homepages of German small animal practices and clinics were included. In order to set the target number of homepages, an estimation based on the data of the Bundestierärztekammer [28] was performed. Because of the improbability of employees owning a veterinary homepage, only self-employed veterinarians were included in this calculation. The target point was set at 10% of all small animal veterinarians, based on the comparable response rate in the study of Hahn et al. [2] for the use of veterinary naturopathy in German-speaking countries (Germany, Switzerland, Austria). For calculating the targeted number of homepages, the distribution of small animal practitioners throughout Germany, and therefore, their percentage within the different Chambers has been included (Table 1).

Data collection was performed using the Windows Internet Explorer, Google Chrome, and Firefox alternately. The search itself was performed with Google. Server history was deleted after each search to avoid algorithms wherever possible. “Veterinarian” and “Chamber of Veterinary Surgeon” (e.g., “veterinarian” “Saxony”/“Tierarzt Tierärztin” “Sachsen”) were used as search items. The Homepages were included from first to last name, until the aimed number was recorded. They were listed with name of practice, postal code, date and time of investigation using Excel 2013 Version 15.0. After the data had been collected, a postcode comparison was performed, avoiding duplicates or classification into incorrect Chambers of Veterinary Surgeons. Afterward data were partly anonymized using continuous numbers.

For data collection the homepages were systemically surveyed for the following information:

- Is there a content of veterinary naturopathy or veterinary complementary medicine? (Yes/No)
- If yes: Which content/treatment modality?
- Is there a stated qualification, regarding to cited treatment modalities (Yes/No)
- If yes: Which one?

All treatment modalities of naturopathy and complementary medicine were included into data collection. Actual offered treatment modalities itself, pictures, terms, and links to homepages of relevant information were included as content. If the contents were apparently offered from non-veterinarians, they were excluded.

Named and traceable further education courses and educational attainments, memberships of research groups and associations were included as qualification. Qualifications listed as “area of interest” or “qualification in …” without stating the explicit name / course and physiotherapists were excluded.

Named and traceable further education courses and educational attainments, memberships of research groups and associations were included as qualification. Qualifications listed as “area of interest” or “qualification in …” without stating the explicit name / course and physiotherapists were excluded.

For the descriptive data analysis, treatment modalities of a former questionnaire study of the authors were used (27) as categories. They based on an extensive literature research in Pubmed, Livio, and Google Scholar, defining the most often cited treatment modalities of veterinary naturopathy and complementary medicine. Treatment modalities known in human medicine and “veterinary” were used as search items, and each bibliography was researched for further possible literature. From this literature review, the following treatment modalities were included and summarized wherever possible: Homeopathy (classic and complex), phytotherapy, TCM (including Chinese herbs and acupuncture), physical treatments (Laser therapy, use of magnetic fields, ultrasound), manual therapy (including osteopathy and chiropractic), diverting therapies (leeches), Bach flower remedies, neural therapy, homotoxicology, and organotherapy [7, 8, 10, 11, 12, 13, 14, 15, 16, 17, 18, 19, 20, 21, 22, 27].

Using these categories, a key word search was performed based on the Excel data. Used key words were (translation in parenthesis):

- “homöo*” (homeo)
- “phyto*” and/or “Pflanzen” (plant)
- “chinesisch*” and/or “TCM” and/or “aku*” (chinese*, acu*)
- “laser*” and/or “magnetfeld*” and/or “ultraschall*” (magnetic field*, ultrasound*)
- “manuell*” and/or “osteopath*” and/or “chiro*”
- “blutegel*” (leech*)
- “bachblüten*” (Bach flower remedies)
- “neural*”
- “homotox*”
- “organo*”

## Results

In total 1,083 homepages were investigated, 40 (3.7%) of them represented veterinary hospitals. Contents of veterinary naturopathy and complementary medicine were identified on 657 (60.7%) homepages. With 91.7% (11/12), the Chamber of Veterinary Surgeons of the Saarland showed the highest numbers of contents (Table 1). The lowest percentage (44%, 22/50) was found in Saxony. For all homepages with relevant contents, homeopathy was named most often (58%, 381/657) and organotherapy was cited least (1.2%, 8/657; Table 2).

Within the 657 homepages with veterinary naturopathic or complementary contents 206 (31.4%) homepages gave information about the qualifications of the practitioner. German specialized veterinarians were named 47 times, these include German official titles of veterinary specialists for acupuncture, homeopathy, biological medicine, or physical therapies (Table 3). Stated qualifications were highly diverse, including international certifications, memberships in specialized societies, qualification as non-medical (veterinary) practitioners ahead of veterinary studies, precisely named continued education courses, and doctoral thesis with naturopathic or complementary content (Table 3). With 52.9% (109/206), courses of continued education in this field were cited most frequently.

## Discussion

With 60.7% of researched homages, a high number of German veterinary homepages cited contents of naturopathy and complementary medicine, with homeopathy cited most frequently. The investigated homepages varied widely in quality, ranging from business card structure to the extent of highly structured homepages with a variety of information.

Using a random query of registered veterinary home­pages prevents the bias of an active survey among the target audience, therefore avoiding the exclusive participation of veterinarians which are particularly interested in this field. However, an influence of web browser algorithms cannot be ruled out. Furthermore, homepages usually provide no demographic data for the targeted audience. Thus, it cannot be said if age, employee status, or practice size alters the targeted audience with the possibility of owning a homepage. These data have not been collected. In addition, some practitioners might offer relevant treatments without mentioning them on their homepage. On the other hand, the actual usage of cited treatment modalities is not traceable with the collected data.

In order to create a reasonable overview, a diverse field of treatment modalities has been categorized. For this, data had to be summarized, allowing a possible loss of information. Especially, the item qualification is difficult to trace. As a high number of homepages stated “field of interest,” these answers did not count into data collection, even if these veterinarians may actually have participated in relevant further education. A vast amount of education and certification possibilities were found, representing very different levels of approval.

60.7% (*n* = 657/1,083) of homepages showed contents of veterinary naturopathy or complementary medicine. The contents of naturopathy and complementary medicine on a veterinary homepage cannot map the actual usage of these treatment modalities, but they can offer an insight into the possible actual usage.

For the usage of complementary medicine by German human patients, Härtel and Volger [25] showed 70% of female and 54% of male patients using these treatments. High numbers of usage are also shown for specialized human departments, such as dentistry or orthopedics [3, 4, 6]. The active participation of specifically interested people could be a reason for the slightly higher numbers of usage in these studies. In the retrospective study of Shmalberg and Memon [7], 39% of 5,195 patients in an American veterinarian clinic (Department of University of Florida) were treated with integrative methods. They defined integrative medicine as the combination of conventional with alternative and complementary medicine [7]. This clinic did not offer homeopathy or chiropractic, which may have led to lower numbers of actual usage. However, dietetics and physiotherapy were included [7]. Lana et al. [18] showed that 76% of the included 254 pet owners use complementary medicine for their oncologically ill pets. And for phytotherapy in the German-speaking countries of Switzerland, Germany, and Austria, Hahn et al. [2] reported 79% of 189 veterinarians using these treatment modalities in their practical work. Comparable data exist in the studies of Ertl [29] and Truls [30], even though they investigated small numbers of veterinarians and Truls [30] included large animal practitioners.

Compared to the questionnaire study of the authors, where 85.4% of all participating veterinarians used naturopathy and/or complementary medicine, the researched homepages showed lower numbers [27]. This might be due to the bias of voluntary participation for the questionnaire study compared to the systematic research of homepages. Furthermore, some homepages may not represent the actual usage of these modalities.

In summary, the percentage of homepages of small animal veterinarians showing contents of naturopathic or complementary medicine is high and roughly comparable to these found by other authors with the restriction of different study designs and differing umbrella terms among authors.

Interestingly, the percentage of contents of veterinary naturopathy and complementary medicine varied widely across the different Chambers of Veterinary Surgeons. 91.7% of all included homepages of the Saarland (11/12) showed relevant contents, whereas only 44% in Saxony did likewise (22/50). No general difference between regions like eastern and western Germany could be found. Sociodemographic data, different contents of university education programs or continuing education programs may influence this distribution. This study cannot offer explanatory data for these differences. In addition, for some Chambers of Veterinary Surgeons with 10%, the number of evaluated homepages was low.

The most frequently cited treatment modality on veterinary homepages was homeopathy with 58% (381/657), followed by TCM with 48.1% (316/657). Complex and classic homeopathy were summarized. Organotherapy (1.2%, 8/657) and homotoxicology (2.9%, 19/657) were found last frequently (Table 2). Prospective, randomized, and double-blinded studies for small animal homeopathy are rare and almost exclusively with dogs as patients [31, 32]. The high numbers of veterinary users could be influenced by a high customer demand, monetary reasons and widely available known products, a positive personal experience could be another reason for usage [11].

In the retrospective study of Shmalberg and Memon [7] of 2015, classic acupuncture was shown to be the most often used integrative treatment option, limiting the fact, that the surveyed clinic did not offer homeopathy. In the study of Baatsch et al. [6] of 2017, over 60% of 250 dentists used phytotherapy and 57% homeopathy. In human medicine, Alscher [3] found that 41.7% of all 935 participants never use homeopathy in their practical work. A survey among owners of pets with oncological diseases, showed homeopathy, phytotherapy, acupuncture, chiropractic, and Bach flower remedies to be used most frequently [18]. Arlt and Heuwieser [1] state acupuncture, phytotherapy, and homeopathy as the three main areas of naturopathic and complementary treatments in veterinary medicine, without giving numbers for this. However, phytotherapy was not one of the top cited treatment modalities in our research. Compared to the usage by human patients [5], it seems reasonable to say, owners seek for treatment modalities for their pets, which they use for themselves. Acupuncture and homeopathy were the most frequently used treatment modalities by human patients in the study of Bücker et al. [5].

Consistent with the results of this study, homeopathy was the most often cited treatment option in the questionnaire study of the authors (27). Interestingly, phytotherapy was used frequently in the questionnaire study whereas it was less frequently cited on the homepages (27). Phytotherapy may not be cited more often because of the widespread use of phytopharmaceuticals within conventional medicine. Therefore, it might not be cited despite its actual usage. Additionally, phytopharmaceuticals are mostly obtainable without prescription, including the risk of delayed indicated medical treatment. On the other hand, this situation is the same for homeopathic remedies. Further data are needed to evaluate these differences.

The research of given qualifications on veterinary homepages with contents of naturopathy and complementary medicine showed diverse qualifications. In total, 31.4% (*n* = 206/657) of all homepages with relevant content gave information about background qualifications. Nevertheless, it is possible that practitioners may not provide information about their qualification online. At the moment, it is not obligatory for veterinarians to give information about their qualifications for offered treatments. This applies for all veterinary medicine treatments. On the contrary in German, human medicine official certification is crucial in order to have these treatments covered by health insurance. Alscher [3] surveyed further certifications for human general, internal, and orthopedic practitioners. They showed 34% of the surveyed group had additional designations for acupuncture, 3.5% for homeopathy, 37.5% for manual medicine, and 10.2% for classic naturopathy [3]. These numbers match the higher numbers of the veterinary additional designations for acupuncture, compared to homeopathy in this study. In the study of Alscher [3], the numbers varied within specialized fields, as e.g., orthopedics had higher numbers for acupuncture possibly due to their indication field. This may be the case in veterinary medicine as well, as specialization is increasing.

The type of qualifications varied broadly. This reflects the given situation for continuing veterinary education in this field. Two main groups can be characterized: veterinary versus human medicine education contents. In addition, short-term courses and long-term education programs exist. International and European certified qualifications exist for acupuncture, chiropractic, or homeopathy (e.g., IVCA/International Veterinary Chiropractic Association, IAVC/International Academy of Veterinary Chiropractic, IVAS/International Veterinary Acupuncture Society, EAVH/European Academy of Veterinary Homeopathy, EAVC/European Association Veterinary Chiropractic, IAVH/International Association for Veterinary Homeopathy, AVCA/American Veterinary Chiropractic Association). In Germany, organizations like GERVAS (German Veterinary Acupuncture Society) or GGTM (Society for Holistic Veterinary Medicine e. V.) offer courses for veterinarians. Furthermore, there are different long-term specialist veterinarian certifications (“Zusatzbezeichnung”; e.g., acupuncture). However, the content and the names of certifications can vary between the Chambers of Veterinary Surgeons. Besides this, a variety of short-term advanced training sessions take place each year. In this study, continued education programs were cited most frequently. There exist courses which are accredited by the ATF (Academy for Veterinary Continuing Education, certifies veterinary continuing education in Germany) and ones which are not ATF-accredited. Furthermore, 19 veterinary practitioners finished a non-medical (veterinary) practitioner education before studying veterinary medicine (Table 3). It was not part of this study to evaluate the whole variety of possible qualifications and the given data can provide only an insight into the actual situation. It can be stated that the vast amount of qualifications may be difficult for pet owners to understand and evaluate.

## Conclusion

The given data illustrate the contents of veterinary naturopathy and complementary medicine on German veterinary homepages. The data show a high percentage of homepages with contents of naturopathy and complementary medicine, comparable to data of high numbers of usage in human and veterinary medicine. Therefore, further scientific research in this field seems reasonable. Modes of treatment and qualifications are highly diverse and despite of controversial public discussions, homeopathy is cited most frequently.

## Statement of Ethics

This study was approved by the Justus-Liebig-University Gießen (Germany). It was part of a doctoral thesis and no ethical permission had to be obtained. Data anonymity was respected and no individual data are traceable.

## Conflict of Interest Statement

None of the authors has any conflict of interest.

## Funding Sources

This study was part of a doctoral thesis and supported by a postgraduate scholarship of the Karl and Veronica Carstens-Foundation.

## Author Contributions

Ines Stanossek: designed the study with the help of Axel Wehrend, data collection, and drafted the manuscript. Both authors read and approved the final manuscript.

## Data Availability Statement

All relevant data are included within this article. For further information, the authors can be contacted due to data anonymity.

## Figures and Tables

**Table 1:** Targeted homepages of small animal veterinarians and their content of veterinary naturopathy and complementary medicine (based on veterinary statistical data of 2016 [28])

Chamber of veterinary surgeons	Self-employed small animal practitioners = A	Self-employed mixed small and large animal practitioners = B	Total = A + B = C	10% of C	Homepages with relevant content, *n* (%)
Baden-Württemberg	566	570	1,136	113.6	72 (63.2)
Bavaria	1,153	704	1,857	185.7	116 (62.4)
Berlin	377	10	387	38.7	20 (51.3)
Brandenburg	216	264	480	48.0	29 (60.4)
Bremen	48	2	50	5.0	3 (60.0)
Hamburg	148	19	167	16.7	9 (52.9)
Hessen	581	365	946	94.6	65 (68.4)
Mecklenburg-Vorpommern	64	166	230	23.0	11 (47.8)
Niedersachsen	695	705	1,400	140.0	87 (62.1)
Nordrhein	763	276	1,039	103.9	53 (51.0)
Westfalen-Lippe	506	347	853	85.3	46 (54.1)
Rheinland-Pfalz	277	246	523	52.3	28 (53.8)
Saarland	74	49	123	12.3	11 (91.7)
Saxony	184	317	501	50.1	22 (44.0)
Sachsen-Anhalt	132	162	294	29.4	16 (55.2)
Schleswig-Holstein	254	293	547	54.7	47 (85.5)
Thüringen	119	167	286	28.6	22 (75.9)
**Total**	6,157	4,662	10,819	**1,083**	**657 (60.7)**

**Table 2:** Results of a key word search on homepages of small animal practitioners for contents of veterinary naturopathy or complementary medicine (multiple answers possible, relative numbers based on 657 homepages with relevant contents in total)

Category/key word; translation	Homepages with relevant content, *n*	Homepages with relevant content, %
Homeopathy (“homöo*”)	381	58.0
Phytotherapy (“phyto*,” “Pflanzen*”)	84	12.8
TCM in total	316	48.1
“chinesisch*,” “TCM”	49	7.5
“aku*”	267	40.6
Biophysical treatments in total	290	44.2
“laser*”	180	27.4
“magnetfeld*” (magnetic field)	105	16.0
“ultraschall*” (ultrasound)	5	0.8
Manual treatments in total	198	30.1
“manuell*”	45	6.8
“osteopath*”	82	12.5
“chiro*”	71	10.8
Diverting treatment/leech (“blutegel*”)	101	15.4
Bach flower remedies (“bachblüten*”)	129	19.6
Neural therapy (“neural*”)	60	9.1
Homotoxicology (“homotoxi*”)	19	2.9
Organotherapy (“organo*”)	8	1.2
Link/reference to colleague/non-medical practitioner	49	7.5

**Table 3:** Qualifications for veterinary naturopathic and complementary treatment modalities, as specified on homepages of small animal veterinarians (relative numbers based on 206 homepages with relevant content in total; multiple answers possible)

Qualification	*n*	%
Specialist veterinarian acupuncture	21	10.2
Specialist veterinarian homeopathy	15	7.3
Specialist veterinarian physical therapies	5	2.4
Specialist veterinarian biological medicine	6	2.9

Certification IVCA^1^	21	10.2
Certification IAVC*	20	9.7
Certification IVAS*	8	3.9
Certification EAVH*	3	1.5
Certification EAVC*	4	1.9
Certification IAVH*	2	1.0
Certification AVCA*	1	0.5
Certification IFAO*	1	0.5
Certification BackBone Akademie	5	2.4
Certification Qi Academie	4	1.9
Certification Tao Equlibre	4	1.9
Certification DIPO*	2	1.0
Certification ABVA*	1	0.5
Certification Akuvett*	1	0.5

Non-medical practitioner	9	4.4
Veterinary non-medical practitioner	10	4.9

Membership GGTM*	34	16.5
Membership DÄGFA*	6	2.9
Membership GERVAS*	6	2.9
Membership AG* Goldimplantation	2	1.0
Membership AG* Lasermedizin	1	0.5
Membership GBM* e. V	1	0.5
Membership DZVhÄ*	1	0.5
Membership AG Klass. Akup. und TCM e. V	1	0.5
Membership ÖGVH*	1	0.5
Membership BEVAS*	1	0.5
Membership DAGC*	1	0.5

Bergische Akademie für Erwachsenenbildung	1	0.5
Continued Education with relevant contents	109	52.9
FTA Chiropraktik (Austria)	4	1.9

Thesis with relevant content	1	0.5

* IVCA, International Veterinary Chiropractic Association; IAVC, International Academy of Veterinary Chiropractic; IVAS, International Veterinary Acupuncture Society; EAVH, European Academy of Veterinary Homeopathy; EAVC, European Association Veterinary Chiropractic; IAVH, International Association for Veterinary Homeopathy; AVCA, American Veterinary Chiropractic Association; IFAO, Institute for applied Osteopathy; DIPO, German Institute for Osteopathy of horses; Akuvett, Acupuncture and Training center for horses and small animals Rimbach; GGTM, Society for Holistic Veterinary Medicine e. V.; DÄGFA, German Medical Society for Acupuncture e. V.; GERVAS, German Veterinary Acupuncture Society; AG, Working Group; GBM, Society for Biochemistry and Molecular Biology e. V.; DZVhÄ, German Central Association of Homeopathic Doctors; ÖGVH, Austrian Society for Veterinary Homeopathy; BEVAS, Belgian Veterinary Acupuncture Society; DAGC, German-American Society for Chiropractic e. V.
